# Clinical Decision-Making Case: Pulmonary Embolism

**DOI:** 10.21980/J8.52339

**Published:** 2025-12-31

**Authors:** James H Lee, Linda Herman

**Affiliations:** * Sutter Roseville Medical Center, Department of Emergency Medicine, Roseville, CA

## Abstract

**Audience:**

Emergency medicine residents and medical students on emergency medicine rotations.

**Introduction:**

Pulmonary embolism (PE) is a common diagnosis with an annual incidence of approximately one in 1000 persons.^1^,^2^,^3^ There is a wide variety of clinical presentations, ranging from the asymptomatic patient to shock and cardiac arrest. Most patients have chest pain and shortness of breath (SOB), but PE may also present with mild or nonspecific symptoms, such as dizziness, cough, wheezing, syncope and hemoptysis. These patients have risk for clinical decompensation.^4^,^5^ It is therefore critical to maintain a high level of suspicion because misdiagnosis is common. There are risks attributable to the diagnostic evaluation and treatment, including radiation exposure, contrast reactions and complications related to anticoagulant therapy. Work up requires an understanding of clinical pretest probability, diagnostic algorithms such as the modified Wells scoring system and the revised Geneva scoring system, the pulmonary embolism rule-out criteria (PERC), and interpretation of D-dimer testing and diagnostic imaging.^6^,^7^ Management requires anticoagulation, but for the unstable patient may also require respiratory and hemodynamic support, systemic or catheter-directed thrombolysis, catheter or surgical embolectomy, or extracorporeal membrane oxygenation (ECMO) if available. Understanding the diagnostic evaluation and management of pulmonary embolism is essential for the practicing emergency medicine physician.

**Educational Objectives:**

By the end of the clinical decision-making case, the learner will: 1) gain familiarity with clinical decision-making (CDM) case format to be used in the new American Board of Emergency Medicine (ABEM) certification examination starting in 2026, 2) demonstrate the ability to obtain a focused history and physical examination and develop appropriate differential diagnoses for chest pain and dyspnea, 3) demonstrate understanding of clinical decisions rules to estimate the pre-test probability for pulmonary embolism and the application of rules to guide appropriate diagnostic testing, 4) recognize high clinical suspicion for pulmonary embolism and indication for empirical treatment, 5) recognize the unstable patient and provide appropriate hemodynamic and respiratory support, 6) understand indications for thrombolytic therapy or embolectomy in unstable pulmonary embolism, 7) demonstrate communication skills with patients and specialists across the health care spectrum, and 8) arrange appropriate disposition for the unstable patient with a pulmonary embolism.

**Educational Methods:**

This session is based on the clinical decision-making (CDM) case format introduced by ABEM to be used in the oral certification examination starting in 2026.^8^ The materials were modeled after the samples provided in the instructional videos on the ABEM Qualifying Exam Part 2 released in December 2024. Slides were provided to the instructor concerning clinical presentation, differential diagnosis, and management for the debrief following the session. This case was tested using 18 resident volunteers ranging from PGY 1–2 in an Accreditation Council for Graduate Medical Education (ACGME) accredited emergency medicine residency program. This was our first mock board session using the CDM format.

**Research Methods:**

Prior to the session, the learner was asked to complete a pre-survey to see if the learner had previously reviewed the ABEM instructional video demonstrating a CDM case. Immediate feedback was then solicited both from the learners and from the evaluators following the debriefing session. Residents were asked to evaluate the educational value of the case using a 1–5 Likert scale (5 being excellent). Evaluators were asked to score the residents by designating whether the learner was able to provide the correct responses concerning required appropriate historical information, physical examination findings, diagnostic testing needed, differential diagnoses, interpretation of diagnostic results, reaching the correct diagnosis, management and disposition of the patient and coordinating transition of care. The examiner would mark the evaluation form with either a yes or no for each response.

**Results:**

Eighteen residents (nine PGY1 residents and nine PGY2 residents) completed the case. We were a new residency program at the time and did not yet have any PGY3 residents. The average score was 28.9 out of 29 points. The pre-survey revealed that only two of the nine EM PGY1 and four of the nine EM PGY2 had reviewed the ABEM video. Eighteen residents completed the post-survey which was done immediately after the simulation. The learners rated the educational value of the case 4.9/5 (5.0/5 for PGY1, 4.9/5 for PGY2). Fifteen residents (8/9 PGY1, 7/9 PGY2) stated that the case increased their understanding of key concepts about the disease process “somewhat,” while three responded that they have had similar patients and did not learn anything new. Thirteen residents (8/9 PGY1, 5/9 PGY2) said that the experience made them more comfortable with the new testing process but that they needed more practice, while only two residents (both PGY2) responded that they were very comfortable with the process.

**Discussion:**

The objective of this oral boards case was two-fold: to give residents experience with the new CDM case format of the ABEM certifying exam and to reinforce the work-up and management of pulmonary embolism.

This simulation was an effective educational tool for residents to gain familiarity with the CDM case portion of the ABEM certifying exam. Only a minority (6/18, 33%) of the residents were familiar with the new testing format prior to the case. This session was the first mock oral board session using a CDM case. Post survey results revealed that 72% of the residents (13/18) said that the experience made them more comfortable with the new testing method but that they needed more practice, while only two residents (11%) stated that they were very comfortable with the process.

This was also a learning opportunity for the examiner in this new CDM case format. The evaluation form used a dichotomous yes/no format which likely contributed to excessive prompting which inflated scoring. This may not accurately reflect the experience at the certifying exam. In response, more specific criteria regarding the degree of prompting and timing of case were added to the script. Repetition of testing in this format should be helpful for residents and educators as preparation for the ABEM certifying exam.

The initial evaluation of pulmonary embolism is a topic with which most residents are comfortable. Residents scored well on testing, suggesting an understanding of the work-up for chest pain and routine management for pulmonary embolism. They had less familiarity with management of the high-acuity, unstable presentation.

This case was not tested on medical students, but we anticipate that this would be an appropriate learning experience for the medical student on an emergency medicine rotation, without need for modification.

This case was designed to introduce residents to the CDM case format. There is limited existing training material for the new oral board exam, and we feel this simulation case is valuable for residents to gain familiarity with the new ABEM certifying exam format through a comfortable topic and a “low pressure” setting.

**Topics:**

Clinical decision-making case, pulmonary embolism, shortness of breath, dyspnea.

## USER GUIDE

**Table:** 

List of Resources:	
Abstract	212
User Guide	215
For Examiner Only	217
Certifying Exam Assessment	226
Stimulus	228
Debriefing and Evaluation Pearls	238

**Learner Audience:**

Medical Students, Interns, Junior Residents, Senior Residents

**Time Required for Implementation:**

Case: Clinical Decision-Making cases are 15 minutes as directed by American Board of Emergency Medicine (ABEM).

Debriefing: 5 minutes

**Recommended number of learners per instructor:**

1 to 3

**Topics:**

Clinical decision-making case, pulmonary embolism, chest pain, shortness of breath, dyspnea.

**Objectives:**

By the end of the clinical decision-making case, the learner will:

1. Gain familiarity with the CDM case format to be used in the new ABEM certification examination starting in 2026.
2. Demonstrate the ability to obtain a focused history and physical examination and develop appropriate differential diagnoses for chest pain and dyspnea.
3. Demonstrate understanding of clinical decisions rules to estimate the pre-test probability for pulmonary embolism and the application of rules to guide appropriate diagnostic testing.
4. Recognize high clinical suspicion for pulmonary embolism and indication for empirical treatment.
5. Recognize the unstable patient and provide appropriate hemodynamic and respiratory support.
6. Understand indications for thrombolytic therapy or embolectomy in unstable pulmonary embolism.
7. Demonstrate communication skills with patients and specialists across the health care spectrum.
8. Arrange appropriate disposition for the unstable patient with a pulmonary embolism.

### Linked objectives, methods and results

This clinical decision-making (CDM) case was chosen to give residents experience with the new ABEM certifying exam format through a familiar, low stress presentation of a straightforward case of pulmonary embolism (objective 1). This case also allows more junior learners to reinforce the history, exam, work-up and initial management of the chest pain patient (objectives 2, 3, 4). As the case progresses, the learner will be challenged to initiate stabilization of the hypotensive patient and consider advanced management with thrombolytics and consultation with specialists (objectives 5, 6, 7, 8).

### Recommended pre-reading for instructor

- Kabrhel, C. Chapter 74: Pulmonary Embolism and Deep Vein Thrombosis. In: Walls R, Hockberger R, Gausche-Hill M, et al, eds. Rosen’s Emergency Medicine - Concepts and Clinical Practice E-Book. 10th ed. Available from: Elsevier eBooks+, Elsevier - OHCE, 2022.
- American College of Emergency Physicians Clinical Policies Subcommittee (Writing Committee) on Thromboembolic Disease: Wolf SJ, Hahn SA, Nentwich LM, Raja AS, Silvers SM, Brown MD. Clinical policy: Critical issues in the evaluation and management of adult patients presenting to the emergency department with suspected acute venous thromboembolic disease. *Ann Emerg Med*. 2018;71(5):e59–e109. doi:10.1016/j.annemergmed.2018.03.006
- American Board of Emergency Medicine. Retrieved from URL: www.abem.org/get-certified/certifying-exam/certifying-exam-content/

### Results and tips for successful implementation

This case is best implemented by following the new CDM case format of the ABEM certifying exam. Examiners should review the ABEM website and videos, the case summary, and the scoring sheet prior to the session. This case was administered to 18 residents as part of our oral board simulation examination series during didactics on 4/24/2025. One faculty instructor served as the examiner and administered the case to each resident individually. Each resident was allotted 30 minutes with the proctor, consisting of 15 minutes for doing the case, five minutes for debrief, and a final five minutes for scoring the resident. Scoring was collected by the examiner with a dichotomous yes/no scale for correct responses concerning required actions for history, physical examination, differential diagnoses, ordering and interpreting of diagnostic studies, diagnosis, management, disposition of the patient and communication during transition of care. We received positive feedback from the residents, rating the educational value of the case 4.9/5. Thirteen residents (72%) said that the experience made them more comfortable with the testing process. Examiner feedback included difficulty with scoring given lack of standardization as to degree of prompting that should be given and the challenges of using a yes/no scale. In response, the case was modified to include more specific criteria regarding degree of prompting and clarification on scoring and critical actions. We found that PGY-1 residents required more prompting to gather historical data for Wells score and PERC criteria. In general, residents required more assistance with the unstable patient and the decision for thrombolysis or surgical or catheter-directed embolectomy. This likely reflects lack of exposure to the critically ill patient with PE and variation in practice patterns and availability of services at their institution. We feel that learners will benefit from this experience, reinforcing their clinical knowledge and gaining experience with CDM case format. This case may also be modified for use as a simulation case.

## Figures and Tables

**Table:** 

**I. History**		Yes	No
**1a**	Essential historical component—Description of chest pain, associated symptoms, past history		
**1b**	Essential historical component—Risk factors for PE/DVT		
**2a**	Rationale for description of chest pain		
**2b**	Rationale for risk factors for PE		
**II. Physical Examination**			
**3a**	Essential physical exam—respiratory		
**3b**	Essential physical exam—cardiovascular		
**4a**	Rationale for lung auscultation		
**4b**	Rationale for leg exam		
**III. Differential Diagnosis**			
**5a**	Differential diagnosis—includes PE		
**5b**	Differential diagnosis—includes ACS/unstable angina/MI		
**5c**	Differential diagnosis—includes one other diagnosis from acceptable list		
**IV. Diagnostic Studies**			
**6a**	Diagnostic studies—ECG		
**6b**	Diagnostic studies—chest x-ray		
**6c**	Diagnostic studies—CT angiogram		
**7a**	Rationale for ECG		
**7b**	Rationale for chest X-ray		
**V. Treatment and Other Actions**			
**8a**	Treatment—anticoagulation		
**8b**	Rationale for anticoagulation		
**9a**	Treatment—managing unstable PE		
**9b**	Contraindications for anticoagulation		
**VI. Final Diagnosis**			
**10**	Diagnosis—massive PE		
**VII. Disposition**			
**11**	Disposition—admission to ICU		
**12**	Rationale for level of care for admitting a patient with PE		
**IX. Transitions of Care**			
**13**	Transition of care—contact admitting and consulting services		
**14**	Transition of care—discuss diagnosis with patient and family		

**Table:** 

**Candidate Task Sheet**	
**#1**	**Emergency Department Admitting Form**
**#2**	**Historical Information**
**#3**	**Physical Exam Findings**
**#4**	**ECG**
**#5**	**Chest x-ray**
**#6**	**Results**
**#7**	**CTA**
**#8**	**Cardiac Ultrasound**

**Table:** Clinical Decision-Making Task Sheet

**CASE PARAMETERS**
This is a 15-minute case You will interact with two examiners. This is an interview style without role playing; you should simply reply to the questions asked. You may be interrupted to move you through the case; this is not a reflection of your performance.
**PATIENT INFORMATION**
Patient’s Age: 49 years Gender: Female Method of Arrival: Private Car Chief Complaint: Chest pain Person Providing History: Patient
**VITAL SIGNS**
BP: 96/54 P: 122 R: 22 T: 37.2° Celsius 02Sat: 92% room air
**TASK STATEMENT**
Your tasks are as follows: List pertinent elements of a focused history and physical exam Develop an appropriate differential and/or provisional diagnosis Select and interpret appropriate studies Articulate appropriate patient management including disposition instructions

**STIMULUS 1:** Emergency Department Admitting Form

**Patient Information**	
**Patient Name**	Frances Ferrari
**Age**	49
**Gender**	Female
**Method of Arrival**	Private auto
**History of Present Illness**	Left-sided chest pain
**Vital Signs on ED Arrival**	BP: 96/54 P: 122 R: 22 T: 37.2° C O2 sat: 92 % on RA

**STIMULUS 2:** Historical Information

**History**
Description of pain: Started 3 hours ago, acute onset, while lying in bed Located on the left side of chest Constant, stabbing, does not radiate Feels short of breath Worse with deep breath or movement Associated symptoms: Mild residual aching pain and swelling of left leg due to recent ACL repair 3 weeks prior Negative for the following: Fever/chills Cough Hemoptysis Recent illness or injury Nausea, vomiting, abdominal pain Dysuria, hematuria
**Past History**
Medical History: Hypertension, hypothyroidism Surgical History: Left ACL repair 3 weeks prior Medications: Lisinopril, levothyroxine Allergies: No known drug allergies Social History: Nonsmoker, rare alcohol, denies drugs Family History: Noncontributory

**STIMULUS 3:** Physical Exam Findings

**Physical Examination**	
**General Appearance**	Well-developed female sitting on the gurney, tachypneic, slightly anxious
**HEENT**	Normal
**Neck**	Normal, no jugular venous distention
**Respiratory**	Tachypneic, increased effort, breath sounds clear
**Cardiovascular**	Tachycardic, regular, normal heart tones, no murmurs, peripheral pulses palpable and symmetrical
**Abdomen**	Normal
**Extremities**	Left lower extremity—healing incisions about the left knee, no erythema or drainage, edema present at knee and calf, tender posterior knee and proximal calf, some decreased flexion at knee due to pain
**Neurologic**	Normal

**STIMULUS 4 f1-jetem-10-5-ce212:**
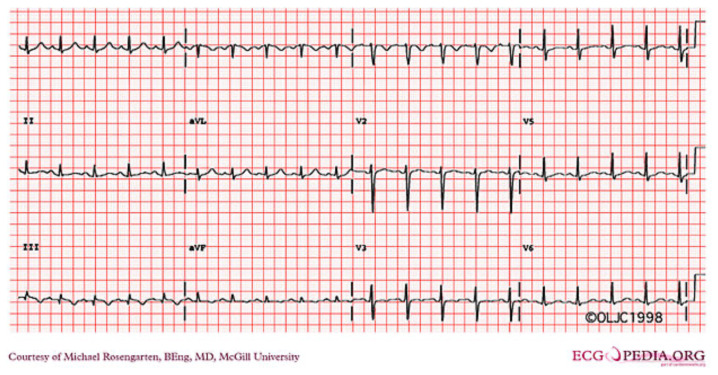
ECG^14^

**STIMULUS 5 f2-jetem-10-5-ce212:**
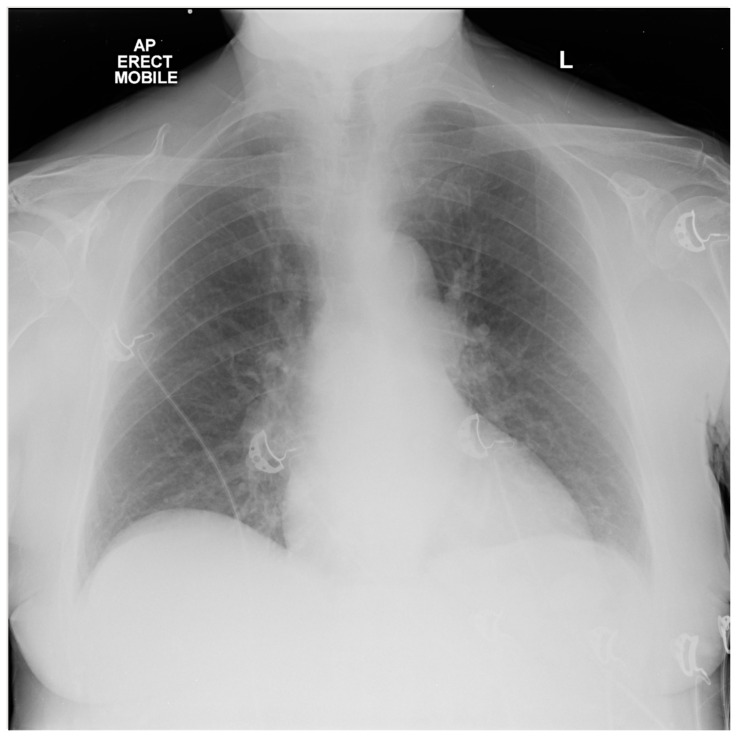
Chest X-Ray^15^

**STIMULUS 6:** Results

CBC	Within normal limits (WNL) except WBC 12.0 × 1000/mm^3^ with normal differential (Normal 4.0–11.0 × 1000/mm^3^)
CMP	WNL
PT, INR, PTT	WNL
Troponin I	167 ng/L (Normal <12 ng/L)
Troponin I (2 hour)	320 ng/L (Normal <12 ng/L)
BNP	762 pg/ml (Normal 100–450 pg/ml)
Creatine Kinase	980 U/L (Normal 22–198 U/L)
CTA Chest	Large embolus located at bifurcation of pulmonary arteries. No infiltrate or effusion.

**STIMULUS 7 f3-jetem-10-5-ce212:**
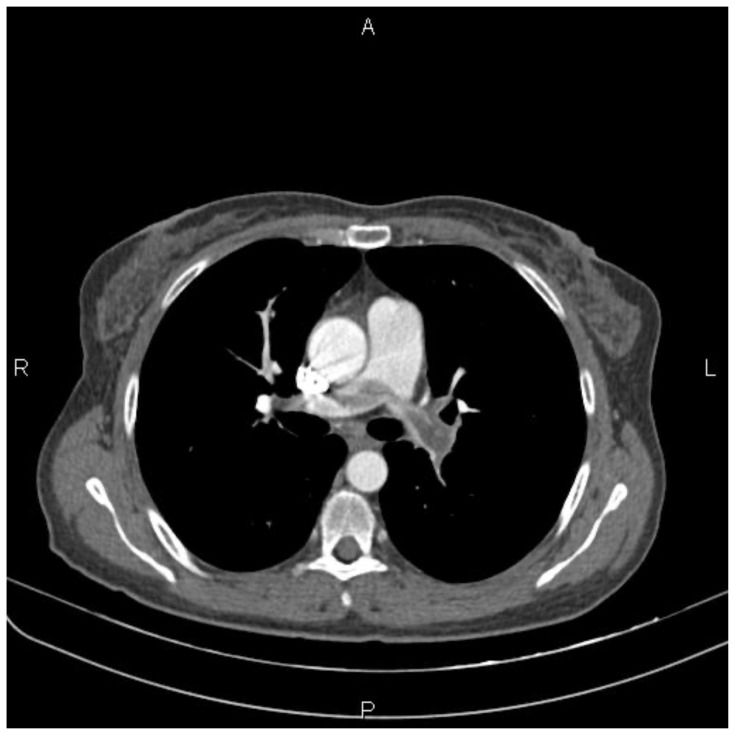
CTA^16^

**STIMULUS 8 f4-jetem-10-5-ce212:**
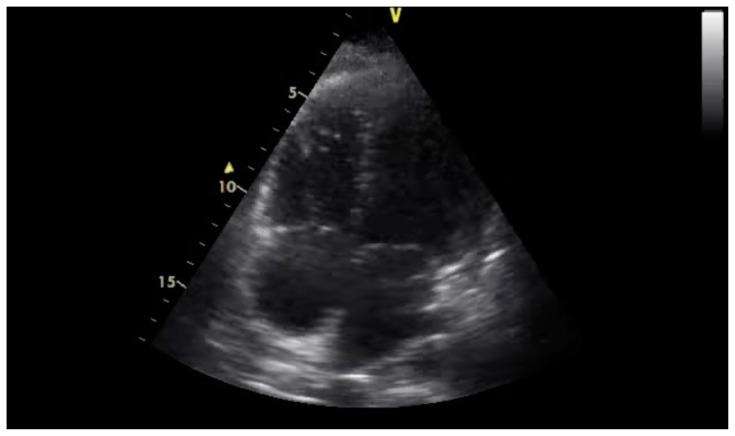
Cardiac Ultrasound^17^

## FOR EXAMINER ONLY

### Clinical Decision-Making Case: Pulmonary Embolism Summary

**Diagnosis:** Pulmonary Embolism

**Case Summary:** The patient is a 49-year-old female, Frances Ferrari, with known history of hypothyroidism and hypertension, an anterior cruciate ligament repair surgery left knee three weeks prior to arrival, who is brought to the ED by family with complaints of three hours of acute onset of left-sided chest pain while lying in bed. The learner should obtain an accurate history and consider pulmonary embolism in the differential. The learner should apply clinical decision rules such as PERC and the modified Wells score to guide further work-up. The learner should initiate diagnostic evaluation for chest pain, including ECG, chest x-ray and troponin. The learner may choose to use the ultrasound for cardiac ultrasound, presence of deep vein thrombosis (DVT), evaluation for pneumothorax, pleural effusion or pneumonia. The learner can order a d-dimer (which will be elevated) or proceed to a computed tomography angiography (CTA) which will show a saddle embolism. The learner should recognize the diagnosis and initiate anticoagulation. The learner should recognize potential for hemodynamic compromise and demonstrate knowledge of supportive interventions. The learner should understand indications for thrombolytic therapy or thrombectomy. The learner should contact the appropriate specialists and arrange ICU level of care. The learner should demonstrate empathetic communication skills with the patient and explain the diagnosis and plan using layman’s language and avoiding jargon.

### Clinical Decision-Making Case: Pulmonary Embolism Examiner Script

**Case Introduction:**

“Hello Doctor, this is a clinical decision-making case. There is no role playing. In response to the questions I will ask, please give me a LIST of information you would gather to come to a final diagnosis. At times, I may interrupt you to move you through the case; this is not a reflection of your performance. You will have 15 minutes to complete the case. Before we begin, do you have any questions?”

“The patient will be a 49-year-old woman who presents with complaints of anterior chest pain which began three hours ago.”

Provide Learner Stimulus #1

#### HISTORY

Prompt 1:

“Here is the initial information regarding this patient. After you have read it, please give me a list of the additional historical information you would obtain.”

The learner should obtain a full pain history; historical critical action includes assessment for PE risk factors.

Information that should be requested:

1. Description of the pain—onset, quality, location, radiation, any aggravating or relieving factors
2. Associated symptoms —fever, cough, shortness of breath (SOB), nausea/vomiting, diaphoresis, lightheadedness, palpitations, neurologic symptoms, extremity pain or swelling, prior history of similar symptoms
3. History:
  Medical illnesses—prior history of chest pain and prior diagnoses of coronary artery disease (CAD), PE, DVT, etc.
  Surgeries—recent surgery increases risk for PE/DVT
  Medications—hormone therapy increases risk for PE/DVT
  Family history—assess risk for PE/DVT, CAD, aortic dissection
  Social history—immobilization/travel may increase risk for PE/DVT, smoking/drugs and risk for CAD
4. Risk factors for pulmonary embolism—previous DVT or PE, recent surgery, fractures, immobilization, clinical signs of DVT, hemoptysis, malignancy, hormone use, family history

Scoring Guidelines:

Learner should elicit history with components from categories 1, 2, 3 and assessment of risk factors from category 4.

General Guidelines:

- If candidate begins managing the case like a standard case, examiner states, “Remember Doctor, there is no role playing in this case. Please list the additional information you want to obtain.”
- If candidate does not offer a complete list of information requested, examiner should pause 10 seconds to allow them to list additional items, before proceeding to the next question.
- If candidate mentions “past medical history,” “family history” or “social history,” the examiner clarifies by asking, “What specifically do you want to know about past medical/family/social history?”

Prompt 2a:

“In a patient with chest pain, why is the description of the chest pain important to you?”

Scoring Guidelines:

Learner should be able to explain that the description of chest pain will aid in determining the differential diagnoses and guide work-up and management.

Prompt 2b:

“In a patient presenting with chest pain, why is asking about risk factors for pulmonary embolism important?”

Scoring Guidelines:

Learner should be able to explain that pulmonary embolism should be considered in this patient and the presence of, or number of, risk factors can be used to estimate risk for pulmonary embolism through clinical decision rules. This will guide diagnostic work-up with a d-dimer or CT angiogram of the chest.

#### PHYSICAL EXAMINATION

“You are provided with the following additional historical information:”

Provide Learner Stimulus #2

Prompt 3:

“Based on what you now know, please give me a list of specific physical examination findings you would be looking for.”

Scoring Guidelines:

The learner should perform a full exam, but certain areas need to be examined; physical exam critical actions are respiratory, cardiac, extremity exams.

Prompt 4a:

“Doctor, why is lung auscultation important and how does it help your evaluation?”

Scoring Guidelines:

Learner should be able to explain that auscultation can help diagnose etiologies such as pneumothorax, pneumonia, pulmonary edema, pleural effusion.

Prompt 4b:

“Doctor, in a patient with chest pain, why is examination of the leg important and how does that help your evaluation?”

Scoring Guidelines:

Learner should be able to explain that extremity exam for tenderness, edema, or signs of infection can suggest diagnoses such as DVT/PE, congestive heart failure (CHF) or sepsis.

#### DIFFERENTIAL DIAGNOSIS

“You are provided with the following physical exam findings:”

Provide Learner Stimulus #3

Prompt 5:

“Based on what you now know, what are the top three items on your differential diagnosis based on the most likely conditions?” (If more than three conditions are mentioned, say, “OK thank you. Please give me your three, and only three, most likely diagnoses.” (If DVT and PE are listed separately, please include them together and request one more diagnosis).

Appropriate differential diagnoses include:

- Pulmonary embolism (PE)/deep venous thrombosis (DVT)
- Acute coronary syndrome (ACS)
- Aortic dissection
- Pneumothorax
- Pneumonia
- Pericarditis/tamponade

Scoring Guidelines:

Critical Action: Differential Diagnosis (DDx)—List must include PE/DVT and ACS and one other diagnosis from the following: aortic dissection, pneumothorax, pneumonia, pericarditis/cardiac tamponade.

#### DIAGNOSTIC STUDIES

Prompt 6:

“Based on what you know and your working differential diagnosis, what, if any, diagnostic studies would you order?”

Scoring Guidelines:

Critical action: diagnostic studies—Learner must order ECG, chest x-ray, CTA chest for PE. If dimer was ordered, without CTA, prompt “The dimer was elevated. Are there any other diagnostic tests you would like to order?”

Other appropriate diagnostic tests may include:

- Metabolic panel
- Complete blood count
- Troponin
- BNP
- Coagulation studies
- D-dimer
- Duplex ultrasound (US)
- Point of care extremity US
- Point of care cardiac US
- Point of care pulmonary US
- Point of care vascular US assessment of aorta or inferior vena cava (IVC)

Prompt 7a:

“Doctor, why would an ECG be important in the evaluation of this patient?”

Scoring Guidelines:

Learner should be able to explain that an ECG may be helpful for PE but is needed to diagnose cardiac ischemia and must include specific discussion of ruling out ST-elevation myocardial infraction (STEMI).

Prompt 7b:

“Doctor, why would a chest X-ray be important in the evaluation of this patient?”

Scoring Guidelines:

Learner should be able to explain that chest x-ray findings such as pneumothorax, pneumonia, mediastinal widening, cardiomegaly or pulmonary edema can help with diagnosis. Explanation must include evaluation for pneumothorax if bedside ultrasound for pneumothorax was not performed.

Provide Learner Stimuli #4, #5, #6, #7, #8

#### TREATMENT AND OTHER ACTIONS

Prompt 8a:

“Based on what you now know, what treatments, if any, would you order and what actions, if any, would you perform?”

Scoring Guidelines:

Critical action—Treatment: Learner must interpret diagnostic tests appropriately and initiate anticoagulation with heparin or low molecular weight heparin. Direct oral anticoagulants (DOAC) should not be used for high-risk pulmonary embolism. It is reasonable for learner to have initiated anticoagulation empirically prior to diagnostic studies based on high suspicion for PE.

Other appropriate actions may include:

- IV access
- Supplemental oxygen
- Crystalloid bolus
- Aspirin therapy
- Point of care ultrasound

Prompt 8b:

“Doctor, why would is heparin or low molecular weight heparin an important treatment in patients such as this?

Scoring Guidelines:

Learner should be able to explain that heparin is an anticoagulant medication that is used as first line treatment for pulmonary embolism and deep venous thrombosis to prevent expansion of thrombus and further embolization.

Prompt 9a:

“If the patient developed worsening hypotension and tachycardia, are there any other additional actions or interventions you would order?”

Scoring Guidelines:

Learner should understand need for further intervention for obstructive shock including fluid bolus and vasopressors. Learner should demonstrate understanding of additional management for unstable massive pulmonary embolism by either initiating thrombolysis or requesting specialty consultation (pulmonary, critical care, interventional radiology, pulmonary embolism response team or cardiothoracic surgery) for recommendations. Additional actions may include bedside hemodynamic monitoring with arterial line, central venous access, and point of care ultrasound vascular and cardiac exams to guide resuscitation.

Prompt 9b:

“Please name three contraindications to thrombolytic therapy.”

Scoring Guidelines:

Learner should be able to describe three of the absolute contraindications listed below:

- Any prior intracranial hemorrhage
- Known structural intracranial cerebrovascular disease (eg, arteriovenous malformation)
- Known malignant intracranial neoplasm
- Ischemic stroke within last 3 months
- Suspected aortic dissection
- Active bleeding
- Bleeding diathesis with coagulopathy or thrombocytopenia
- Recent surgery encroaching on the spinal canal or brain
- Recent closed-head or facial trauma with radiographic evidence of bony fracture or brain injury
- Severe uncontrolled hypertension

#### FINAL DIAGNOSIS

Prompt 10:

“Based on everything you know about this case, what is your final diagnosis?”

- Pulmonary embolism

Scoring Guidelines:

Rationale: Verbalizing PE meets the critical action. If the candidate mentions something vague as diagnosis such as “chest pain,” examiner should prompt once, “Can you be more specific about the diagnosis?”

#### DISPOSITION

Prompt 11:

“Based on what you know, what should be the disposition of this patient?”

Critical action: Disposition—Admission to the ICU.

Prompt 12:

“How do you decide if you should admit a patient with PE to the telemetry ward or the ICU?”

Scoring Guidelines:

Learner should be able to explain that the PE patient with hypotension and tachycardia needs close monitoring for potential respiratory failure requiring intubation or hemodynamic compromise requiring cardiovascular support.

#### TRANSITION OF CARE

Prompt 13:

“What specific actions would you take at the time of admission?

If family, consultants, or admission team were not contacted, prompt:

Prompt 14:

“Is there anyone else you would like to speak with?”

Scoring Guidelines:

Learner should contact all consulting and admitting services and explain the diagnosis to the patient and family.

*Thank you, Doctor. That concludes this case.*

*Please tear up your notes.*

## CERTIFYING EXAM ASSESSMENT

*Clinical Decision-Making Case: Pulmonary Embolism*

Learner: _________________________________________

**Summative and formative comments:**

### Stimulus Inventory

[Fig f1-jetem-10-5-ce212] [Fig f2-jetem-10-5-ce212] [Fig f3-jetem-10-5-ce212] [Fig f4-jetem-10-5-ce212]

## DEBRIEFING AND EVALUATION PEARLS

### Clinical Decision-Making Case: Pulmonary Embolism

- **Testing strategies:** Unlike previous oral board exams that used role playing, the CDM case format involves open-ended questions for the learner to request history and physical exam findings to various clinical vignettes.^8^ Be systematic and use your favorite mnemonic such as OLDCARTS or OPQRST to obtain a complete history. Keep in mind all potentially life-threatening causes and seek out historical and exam findings that may help guide your work-up.
- **History and physical exam:** While this case may seem quite straightforward for PE, most patients present less classically. A thorough history and physical allows determination of a patient’s risk for PE using the Wells or PERC scores.^9^–^11^ Including the elements to calculate these scores is an essential part of the history and physical.
- **Differential diagnosis:** It is important initially to keep your differential diagnosis broad for chest pain and dyspnea. Acute coronary syndrome (ACS) is very common and must be considered for all patients. Older adults, diabetics, and women are more likely to present with less classical symptoms such as shortness of breath, nausea, or other nonspecific symptoms. Gauge risk for ACS with a good history and physical and use of a clinical risk calculator such as the HEART score.
- **Risk stratification:** Many validated decision-making tools have been developed to guide work-up. Age-adjusted dimer increases specificity without modifying sensitivity in patients aged 50 or more with a non-high clinical probability.^12^ The YEARS algorithm may be used for pregnant patients.^13^ Diagnostic studies expose patients to radiation and risks of IV contrast, so choose wisely and use your clinical decision scores to guide workup.
- **Management:** While most patients with PE present clinically stable and treatment is simply anticoagulation, patients with massive PE may present hemodynamically unstable, or even in cardiac arrest. Management of hemodynamic instability or respiratory failure must be concurrent with specialty consultation and consideration of thrombolysis or interventional strategies, based on availability at your institution. It is key to understand the entire spectrum of clinical presentations and the variety of management options for the unstable patient with massive PE.
- **Teamwork:** The critically ill patient with PE may require input from a multidisciplinary team, so early specialty consultation is recommended.
